# More than a Rumor Spreads in Parkinson's Disease

**DOI:** 10.3389/fnhum.2016.00608

**Published:** 2016-12-02

**Authors:** Natalia C. Prymaczok, Roland Riek, Juan Gerez

**Affiliations:** ^1^Laboratorio de Neurofisiología del Instituto Multidisciplinario de Biología Celular, Argentine Research Council (CONICET), National University of La Plata and Scientific Research Commission, Province of Buenos Aires (CIC-PBA)La Plata, Buenos Aires, Argentina; ^2^Laboratory of Physical Chemistry, D-CHAB, ETH ZurichZurich, Switzerland

**Keywords:** Parkinson's disease, alpha-Synuclein, neurodegeneration, cell-to-cell propagation, Lewy bodies, prion-like diseases

## Abstract

As Parkinson's disease progresses, a massive loss of dopaminergic neurons is accompanied by accumulation of alpha-Synuclein (αSyn) neuronal inclusions called Lewy bodies and Lewy neurites. Inclusions first appear in olfactory bulb and enteric neurons then in ascendant neuroanatomical interconnected areas, and finally, in late stages of the disease, Lewy bodies are observed in a substantia nigra pars compacta with clear signs of neuronal loss. It is believed that the spreading of Lewy bodies through the nervous system is a consequence of the cell-to-cell propagation of αSyn, that can occur via sequential steps of secretion and uptake. Certain pathological forms of transmitted αSyn are able to seed endogenous counterparts in healthy recipient cells, thus promoting the self-sustained cycle of inclusion formation, amplification and spreading, that ultimately underlies disease progression. Here we review the cell-to-cell propagation of αSyn focusing on its role in the progression of Parkinson's disease.

## Alpha-synuclein and parkinon's disease

Parkinson's disease (PD) is a complex degenerative disorder that is pathologically characterized by a massive loss of dopaminergic neurons in the substantia nigra pars compacta (SNpc) and the progressive accumulation of Lewy bodies and Lewy neurites (LBs/LNs), two forms of inclusions rich in filaments of aggregated alpha-Synuclein (αSyn) (Spillantini et al., 1997). Although a causative role remains to be formally established, the facts that LBs/LNs are present in virtually all sporadic and familial forms of PD (Poulopoulos et al., 2012), that point mutations and multiplications of the αSyn-encoding gene, *SNCA*, lead to early onset PD (Polymeropoulos et al., 1997; Krüger et al., 1998; Singleton et al., 2003; Chartier-Harlin et al., 2004; Zarranz et al., 2004) and that *SNCA* polymorphisms positively correlate with PD risk (Satake et al., 2009; Simón-Sánchez et al., 2009; Edwards et al., 2010) attest an irrefutable link between PD and αSyn.

Since the discovery that αSyn is abundant in LBs in the late 90s, a tremendous effort has been made to determine the precise 3D conformations adopted by this protein under physiological conditions. It is clear now that in aqueous solution αSyn behaves as an intrinsically disordered protein, lacking a defined or stable structure (Uversky and Eliezer, 2009; Drescher et al., 2012). Although still a matter of extensive debate, an emerging consensus indicates that within healthy cells αSyn exists as soluble low molecular weight species that play important roles in intra and extracellular vesicle trafficking and dynamics (Burre et al., 2010; Bartels et al., 2011; Fauvet et al., 2012; Theillet et al., 2016). In disease-related contexts, however, αSyn is also found as β-sheet-enriched amyloid aggregates that reside within and constitute the building blocks of LBs/LNs (Spillantini et al., 1997; Baba et al., 1998; Conway et al., 1998, 2000). Compelling evidence indicates that the culprits of toxicity are oligomers and higher order assemblies of αSyn such as amyloid fibrils (El-Agnaf et al., 1998; Winner et al., 2011; Rockenstein et al., 2014).

## The cell-to-cell transmission of αsyn

**Figure 1:**
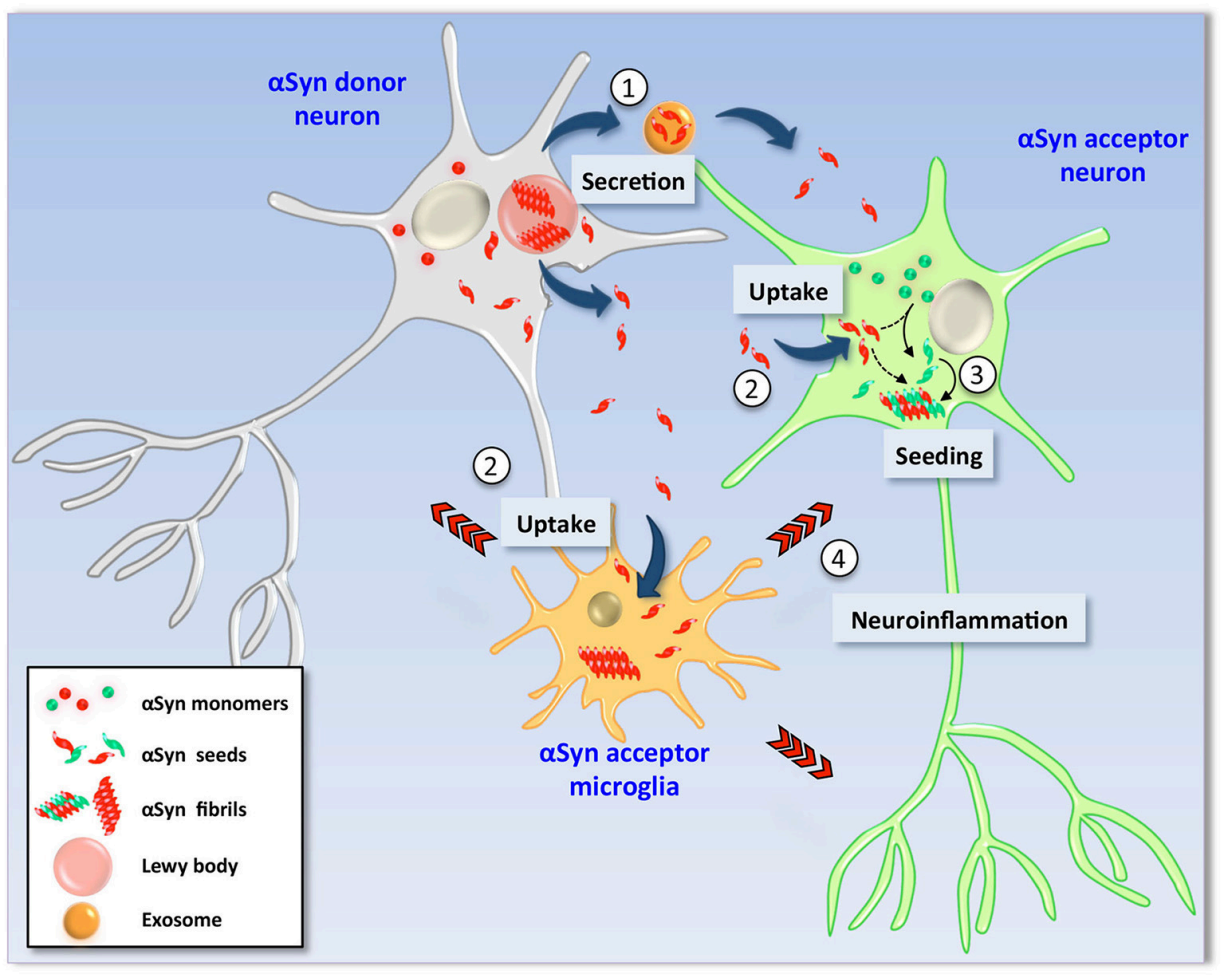
**Hypothetical model of α-Synuclein cell-to-cell transmission**. In pathological conditions, αSyn is found as β-sheet-enriched amyloid aggregates and fibrils that reside within Lewy bodies and Lewy neurites (LBs/LNs). Neurons containing LBs (left) could release αSyn aggregates and seeds into the extracellular milieu by different mechanisms such as non-classical exocytosis or via exosomes (1). Extracellular αSyn is then internalized by endocytosis by neighbor neurons as well as glial cells (2). Due to its amyloidogenic nature, uptake of exogenous seeds promotes the structural corruption of endogenous counterpart in healthy recipient neuronal cells (right). Thus, monomers of the recipient cell are converted into aggregates and fibrils by direct action of exogenous seeds (3) and new LBs are formed (not shown). Neuronal viability is severely affected by two mechanisms; (i) the intrinsic cytotoxic properties of intracellular αSyn aggregates and (ii) indirectly by action of proinflammatory molecules released by glial cells activated upon exposure to extracellular αSyn seeds (4).

Early neuroanatomical studies conducted mainly by Braak and co-workers revealed that LBs appear first in the olfactory bulb and enteric neurons and that only after several years they are found in certain areas of the midbrain such as SNpc and eventually neocortex (Wakabayashi et al., 1988; Braak et al., 2003a, 2006; Braak and Del Tredici, 2008). Thus, during the progression of the disease, LBs are found in a stereotypical and topographical distribution in the nervous system. This highly predictable pattern of LB distribution was not taken into deep consideration until the subsequent discovery (in 2008) that healthy neurons would acquire LBs when grafted into the brains of PD patients (Kordower et al., 2008a,b; Li et al., 2008, 2010). A few years later, the demonstration that αSyn is transmitted from cell-to-cell led to the unifying hypothesis that the transcellular transmission of certain forms of αSyn underlies LB pathogenesis and spreading, and by extension, PD progression (Dunning et al., 2012). This hypothesis was originally supported by clinical evidence suggesting “host-to-graft” transmission of pathological αSyn forms: when embryonic mesencephalic neurons were grafted into PD patient's brains, they developed LBs several years after grafting (Kordower et al., 2008a,b; Li et al., 2008, 2010). The *in vitro* evidence supporting the cell-to-cell propagation of αSyn is its release by unconventional secretion (Emmanouilidou et al., 2010) and the uptake of extracellular αSyn (both natural and recombinant forms) by active mechanisms involving endocytosis (Figure 1; Sung et al., 2001; Liu et al., 2007; Lee et al., 2008a). αSyn can also be transmitted trans-synaptically and through tunnel-like structures that connect the cytosol of neighbor cells (Danzer et al., 2011; Abounit et al., 2016). *In vivo* evidence includes the slow but persistent acquisition of LB-like inclusions by healthy neuronal cells that have been grafted into the brains of mice predisposed to develop LB-pathology spontaneously, such as αSyn transgenic mice (Desplats et al., 2009; Hansen et al., 2011). Similarly, an early onset and widespread LB-like pathology is observed in animals that had received an intracerebral dose of brain homogenates of diseased αSyn transgenic mice (Luk et al., 2012b). While other factors present in brain homogenates could be involved, αSyn alone is sufficient to initiate LB-like pathology and its subsequent spreading: a single intracerebral injection of synthetic αSyn preformed fibrils leads to pathogenesis and progressive accumulation LB-like inclusions in neuroanatomically-interconnected areas accompanied by pathological features of PD such as neurodegeneration, neuroinflammation and motor deficits (Luk et al., 2012a; Sacino et al., 2014). This induction of LB-like pathology by intracerebral administration of αSyn aggregates strictly depends on the presence of αSyn in the host recipient cell, as no pathology can be induced in αSyn knock out mice (Luk et al., 2012b; Mougenot et al., 2012). The requirement of endogenous αSyn on LB-like inclusion spreading is explained by the observation in cell cultures that upon uptake, preformed αSyn fibrils promote the structural corruption of endogenous αSyn and its recruitment into newly formed inclusions (Luk et al., 2009; Waxman and Giasson, 2010; Volpicelli-Daley et al., 2011, 2014). Thus, host αSyn would be essential for the amplification of inclusions, an idea that needs to be further challenged (Helwig et al., 2016). Recruitment of host αSyn by exogenous αSyn has also been demonstrated *in vivo* in most mouse models in which LB pathogenesis is induced by administration of exogenous αSyn seeds (Luk et al., 2012b).

## Braak's hypothesis in PD progression

Braak and colleagues discovered that in sporadic PD, Lewy body pathology is first observed in the lower brainstem and anterior olfactory structures and that it then ascends following a caudo-rostral pattern from the dorsal motor nucleus through susceptible areas of the medulla, pontine tegmentum, midbrain, basal forebrain, reaching in some extreme cases the cerebral cortex (Braak et al., 2003a). These observations elegantly detailed LB spreading along the central nervous system (CNS), but they did not explain where and how the inclusions are originated. Thus, the provocative idea that LB-pathology begins when a neurotropic pathogen enters the nervous system and then spreads in a retrograde-axonal and transneuronal manner from one vulnerable brain region to the next was then introduced (Braak et al., 2003b; Hawkes et al., 2007). While in transit, this pathogen induces formation of αSyn-positive inclusions in these traceable CNS areas. It is now evident that certain species of αSyn can fulfill the requirements for Braak's neurotropic pathogen, and that both the olfactory and gastric tracks are largely compatible with the putative entry routes. In support of this, LB pathology is found in both the anterior olfactory nucleus as well as olfactory bulb mitral cells, the projection neurons that receive inputs from the olfactory epithelium (Daniel and Hawkes, 1992; Braak et al., 2003a). LB pathology has long been known to occur in the gastrointestinal tract of PD patients and is well documented in all the stages of PD (Braak et al., 2006; Lebouvier et al., 2008; Pouclet et al., 2012). Importantly, Braak and colleagues also provided the explanation for the observed non-random distribution of LBs along the nervous system by showing that different cell types have different susceptibilities to the development of inclusions. They concluded that neurons with a long, thin and poorly myelinated axon are highly susceptible to develop inclusions (Braak and Braak, 2000; Braak et al., 2003b). Importantly, these features are found in the enteric vagal preganglionic neurons that are susceptible to developing LB-pathology in early asymptomatic stages of the disease (Braak and Del Tredici, 2004).

## Mechanisms of toxicity

Like many aspects of its intercellular transmission, very little is known on the mechanisms of toxicity inherent to cell-to-cell transmitted αSyn. Likewise, how αSyn transmission is modulated or whether PD-related familial mutations or somatic copy number variations of the *SNCA* gene influence αSyn transmission remains to be determined. Taking into consideration our current knowledge on the consequences of αSyn misregulation, it is conceivable that cell-to-cell transmitted and endogenous αSyn share cytotoxic mechanisms that directly impact neuronal survival. These include, but are not limited to, the loss of function of endogenous αSyn as a consequence of its seeded aggregation, a phenomenon that massively affects neuronal physiological processes such as vesicle trafficking including neurotransmitter release and recycling (Jenco et al., 1998; Abeliovich et al., 2000; Murphy et al., 2000; Cabin et al., 2002; Chandra et al., 2005), the impairment of mitochondrial activity that perturbs not only a plethora of metabolic processes but also degradative pathways (Martin et al., 2006; Devi et al., 2008; Liu et al., 2009; Chinta et al., 2010; Loeb et al., 2010), and the disruption of vesicular transport mechanisms, in particular those that trigger endoplasmic reticulum stress (Cooper et al., 2006; Gitler et al., 2008; Thayanidhi et al., 2010). However, it is still possible that cell-to-cell transmitted αSyn has its own particular repertoire of cytotoxic properties, in addition to its probable distinct physiological functions. The fact that cell-to-cell transmitted αSyn is released to the extracellular milieu allowed neuroscientists to develop cellular and animal models based in administration of exogenous αSyn species that would recapitulate key mechanistic aspects of αSyn transmission such as its internalization and downstream events. Data obtained from glial cells might constitute the first evidence that endogenous and exogenously acquired αSyn might behave differently. It is well known that glial cells normally do not express αSyn mRNA (Reyes et al., 2014) and instead acquire the protein from the extracellular milieu (Liu et al., 2007; Lee et al., 2008b, 2010; Park et al., 2009). Thus, uptake of exogenous αSyn not only would explain the source of αSyn in glial cytoplasmic inclusions (GCIs) in multiple system atrophy (MSA), a progressive neurodegenerative disease related to PD and other synucleinopaties (Tu et al., 1998), but also would uncover a unique role for extracellular αSyn in the context of αSyn-deficient cells. Furthermore, it was shown that extracellular αSyn activates astrocytes and microglia *in vitro* and *in vivo* resulting in a neuroinflammatory response reminiscent to that observed in PD (Zhang et al., 2005; Lee et al., 2010; Alvarez-Erviti et al., 2011; Halliday and Stevens, 2011; Luk et al., 2012a). Noteworthy, neurons are highly susceptible to glial-derived proinflammatory factors, therefore representing an alternative neurotoxic process triggered specifically by cells that have acquired αSyn from the extracellular milieu. Although the neuron-glia interaction might help to elucidate specific and non-redundant roles for intracellular and extracellular cell-to-cell transmitted αSyn, the molecular and biochemical determinants that presumably make these two forms of αSyn different remain completely unexplored.

## The prion hypothesis

As a consequence of Braak's model formulation, the idea that PD behaves as a prion disease has emerged, leading some to refer to it as a “prion-like” disorder. Of note, unlike prions, transmissibility between individuals of pathological forms of αSyn has not been demonstrated and thereby αSyn is currently considered as a non-infectious protein (Aguzzi and Rajendran, 2009; Beekes et al., 2014). The analogy to prion diseases stems for the fact that in *in vivo* experiments, involving mostly rodents and in some cases non-human primates, intracerebral administration of exogenous αSyn (either αSyn-containing brain material or synthetic αSyn proteins) is sufficient to trigger LB pathogenesis, amplification and spreading. These processes are usually accompanied by the phenotypic changes naturally observed in PD such as neurodegeneration, neuroinflammation, and motor deficits. Furthermore, it has been shown that in some cases the particular structural conformation of the exogenous αSyn seed, normally referred to as the “conformational strain”, is transmitted from the exogenously administered aggregates to host αSyn and thereby to the newly formed inclusions (Bousset et al., 2013; Guo et al., 2013; Peelaerts et al., 2015). This supports the idea of that the applied exogenous αSyn acts as seeds that template the aggregation of homotypic molecules of the host, a phenomenon characteristic of prions. The evidence of PD as a prion-like disorder is accumulating, however, there are still several unsolved questions that should be addressed before this terminology is broadly accepted. These questions arise from the inherent limitations of animal models for the full recapitulation of the human condition. In this sense, a constantly growing effort is being made to better characterize the brain material that contains neurotoxic αSyn species and the synthetic αSyn aggregates that are administrated intracerebrally to trigger PD-like pathology (Bousset et al., 2013; Tuttle et al., 2016). To uncover the molecular similarities and differences between the αSyn used in such *in vivo* experiments and those contained in Lewy bodies and Lewy neurites is critical to comprehensively understand the scopes and limitations of such animal models and the resultant hypotheses. As an example of such a gap of information, it has long been reported that certain forms of αSyn are found in the cerebrospinal fluid (CSF) of both healthy subjects and PD patients (Borghi et al., 2000; Tokuda et al., 2006; Mollenhauer et al., 2010; Parnetti et al., 2011; Foulds et al., 2012). However, it remains an enigma which structural species of αSyn are found in CSF and whether these molecules correspond to the transmitted species that mediate the pathogenic process that underlies LB pathogenesis and spreading. Elucidating this will help to reconcile clinical evidence arguing against the concept of prion-like progression in Parkinson's disease and related synucleinopaties (Hallett et al., 2014).

## Concluding remarks

The discovery of the cell-to-cell propagation of αSyn and in particular its role as mediator of disease progression has opened new therapeutic avenues for the treatment of PD and related neurological disorders, and novel therapies targeting extracellular αSyn aimed to delay or stop disease progression are currently being explored. The therapeutic potential of passive immunotherapy targeting aberrant forms of αSyn, for instance, has recently been investigated showing that it efficiently interferes with uptake of extracellular αSyn seeds preventing downstream effects such as amplification and transmission of pathological aggregates (Tran et al., 2014). Similarly, administration of rationally engineered antibodies robustly promotes degradation and neutralization of internalized αSyn preventing cell-to-cell aggregate transmission and neuronal loss (Bae et al., 2012; Spencer et al., 2014). Nevertheless, the elucidation of the mechanisms involved in αSyn transcellular transmission will be instrumental not only for the development of novel therapies for PD but also for the understanding of the “prion-like” properties of amyloid-beta (Aβ), tau and Huntingtin, all of them transmissible aggregation-prone proteins with a long history in neurodegenerative diseases such as Alzheimer and Huntington's diseases, respectively (Brettschneider et al., 2015).

## Author contributions

NP, RR, and JG carried out literature searches, assisted in generation of figures and writing of the manuscript. All authors read and approved the manuscript.

### Conflict of interest statement

The authors declare that the research was conducted in the absence of any commercial or financial relationships that could be construed as a potential conflict of interest.
